# Risk factors for severe rash with use of vemurafenib alone or in combination with cobimetinib for advanced melanoma: pooled analysis of clinical trials

**DOI:** 10.1186/s12885-020-6659-0

**Published:** 2020-02-27

**Authors:** Ashley M. Hopkins, Akash D. Rathod, Andrew Rowland, Ganessan Kichenadasse, Michael J. Sorich

**Affiliations:** College of Medicine and Public Health, Flinders University, Adelaide, 5042 Australia

**Keywords:** BRAF inhibitor, Vemurafenib, Severe rash, Toxicity, Predictors, Risk factor

## Abstract

**Background:**

Rash is one of the most common severe adverse events associated with use of vemurafenib for the treatment of melanoma, either as monotherapy or in combination with cobimetinib. The study aimed to identify pre-treatment patient characteristics predictive of developing severe rash with vemurafenib therapy.

**Methods:**

This was a secondary pooled analysis of individual patient data from the BRIM-2, BRIM-3 and coBRIM clinical trials, including all patients treated with vemurafenib alone and vemurafenib plus cobimetinib. Patient age, sex, performance status, body weight, body mass index, liver function markers and estimated glomerular filtration rate were assessed for association with development of severe (grade 3 or 4) rash using logistic regression.

**Results:**

Of 962 patients treated with vemurafenib, 150 (16%) patients experienced severe rash. Female sex was identified as a significant risk factor for severe rash development (*P* < 0.001), having a two-fold increased risk compared to males (22% vs 11%, odds ratio [OR] 2.17; 95% CI 1.52 to 3.09). Low body weight was also associated with increased risk of severe rash (*P* = 0.002), but this association was not significant after adjustment for sex. The association between sex and risk of severe rash was consistent across clinical trials and treatments (vemurafenib monotherapy, vemurafenib plus cobimetinib).

**Conclusion:**

Females had approximately two-fold increased risk of developing severe rash compared to males in clinical trials of vemurafenib alone or in combination with cobimetinib.

## Background

The BRAF inhibitors, vemurafenib and dabrafenib, have been demonstrated to significantly improve survival outcomes in advanced melanoma [1, 2]. The combination of a BRAF inhibitor with a MEK inhibitor (vemurafenib plus cobimetinib, dabrafenib plus trametinib, encorafenib plus binimetinib) further improves survival outcomes over BRAF inhibitor treatment alone [3–5]. There are no direct comparisons of BRAF-MEK inhibitor combinations, but all three options appear to have similar efficacy and therefore consideration of toxicity profiles is important with respect to treatment selection and monitoring. Although there are similarities in the overall profile of adverse events across BRAF-MEK inhibitor combinations, there are also considerable differences in relative incidence of specific adverse events [1–4].

Skin toxicities such as skin rash (e.g. erythema, maculopapular rash, folliculitis, keratosis pilaris like eruption), photosensitivity, keratoacanthoma and cutaneous squamous cell carcinoma, are collectively the most common severe toxicities associated with vemurafenib treatment [6–10]. Other notable severe toxicities include arthralgia and increase in liver enzymes [9, 10]. In the coBRIM study which compared vemurafenib monotherapy with vemurafenib plus cobimetinib, rash was the most commonly reported severe adverse event for both study arms, and the adverse event most commonly leading to the need for vemurafenib/cobimetinib discontinuation, interruption or dose reduction [3, 10]. Notably, the addition of cobimetinib to vemurafenib markedly reduced the risk of many skin toxicities including squamous cell carcinoma and keratoacanthoma, but not rash [3, 10]. Little is known about the risk factors for vemurafenib-induced severe rash, and thus the aim of this study was to identify pre-treatment patient characteristics that predict the risk of severe rash with use of vemurafenib (alone and in combination with cobimentinib) for treatment of advanced melanoma.

## Methods

This study was a secondary pooled analysis of individual-participant data on adults with advanced BRAF V600 mutation–positive melanoma that participated in the BRIM-2 (NCT00949702), BRIM-3 (NCT01006980) and coBRIM (NCT01689519) clinical trials [1, 3, 11]. BRIM-2 was a phase 2 single arm study of vemurafenib (960 mg twice a day) monotherapy in previous treated patients [11], BRIM-3 was a phase 3 randomised trial that evaluated the first-line use of dacarbazine compared to vemurafenib (960 mg twice a day) monotherapy [1], and coBRIM was a phase 3 randomised trial that compared the first-line use of vemurafenib (960 mg twice a day) monotherapy and the combination of vemurafenib (960 mg twice a day) with cobimetinib (60 mg once a day for 21 days, followed by 7 days off) [3]. Secondary analysis of participant-level data for the BRIM-2, BRIM-3 and coBRIM clinical studies was approved by the Southern Adelaide Clinical Human Research Ethics Committee (SAC HREC EC00188) and accessed according to Roche’s data sharing policy [12]. All participants treated with vemurafenib monotherapy or vemurafenib plus cobimetinib combination therapy, were included in the analysis.

All 3 clinical trials used NCI CTCAE (Common Terminology Criteria for Adverse Events) version 4.0 to report adverse events. The outcome was skin rash of any kind (as per previously defined terms [1, 10]) that occurred while on therapy or within 28 days of discontinuing therapy. The primary outcome measure was severe (grade 3 or 4) rash, and the secondary outcome was rash classified as a serious adverse event (life threatening, requiring/prolonging hospitalization, leading to permanent impairment/damage, or requiring intervention to prevent permanent impairment/damage [13]).

The covariates were pre-selected based on data availability, prior studies and biological plausibility. The factors considered for the analysis included patient age, sex, ECOG performance status, body weight, body mass index (BMI), estimated glomerular filtration rate (eGFR), total bilirubin, aspartate aminotransferase (AST), alanine aminotransferase (ALT) and history of atopy. Continuous variables were categorised by standard cut-points (age, BMI, eGFR, bilirubin, AST, ALT), or otherwise as quartiles (body weight). History of atopy was defined by documented allergic reactions / hypersensitivity, atopic dermatitis, eczema, asthma, rhinitis, antihistamine use, or asthma medications.

Relationships between potential predictive factors and rash were initially assessed using univariate logistic regression (Wald test), with effect size reported as an odds ratio (OR). Covariates with a *P*-value < 0.05 were evaluated using multivariable logistic regression. All analyses were adjusted for study (BRIM-2, BRIM-3, coBRIM) and treatment (vemurafenib monotherapy vs vemurafenib plus cobimetinib). All tests were two-tailed with a significant *P*-value threshold of 0.05. All the statistical analyses were performed using R (version 3.4).

## Results

In total, 962 study participants across the three clinical trials were treated with either vemurafenib monotherapy (*n* = 715) or vemurafenib plus cobimetinib (*n* = 247). Baseline characteristics of the study participants are summarised in Table 1. Of the 962 pooled study participants, 150 (16%) experienced on-therapy severe rash, and 21 (2.2%) experienced rash classified as a serious adverse event. Incidence of severe rash was similar between studies: BRIM-2 (17%), BRIM-3 (13%), and coBRIM (vemurafenib monotherapy: 16%, vemurafenib plus cobimetinib: 17%). Median time to severe rash was 11 days and 90% of events occurred within the first 5 weeks of therapy.

**Table 1:** Summary of patient characteristics

	Total *n* = 962	BRIM2 *n* = 132	BRIM3 *n* = 337	coBRIM *n* = 493
Treatment				
Vemurafenib monotherapy	715 (74%)	132 (100%)	337 (100%)	246 (50%)
Vemurafenib + cobimetinib	247 (26%)	0 (0%)	0 (0%)	247 (50%)
Sex				
Male	565 (59%)	81 (61%)	200 (59%)	284 (58%)
Female	397 (41%)	51 (39%)	137 (41%)	209 (42%)
Age (years)				
Median (IQR)	55 (45–65)	52 (40–63)	56 (47–65)	55 (45–66)
Race				
White	923 (96%)	130 (98%)	333 (99%)	460 (93%)
Other	14 (1%)	2 (2%)	4 (1%)	8 (2%)
Missing	25 (3%)	0 (0%)	0 (0%)	25 (5%)
BRAF V600 mutation				
V600E	762 (79%)	122 (92%)	296 (88%)	344 (70%)
V600K	98 (10%)	10 (8%)	33 (10%)	55 (11%)
Missing	102 (11%)	0 (0%)	8 (2%)	94 (19%)
Stage				
Unresectable IIIc	54 (6%)	0 (0%)	20 (6%)	34 (7%)
M1a	146 (15%)	33 (25%)	33 (10%)	80 (16%)
M1b	162 (17%)	18 (14%)	62 (18%)	82 (17%)
M1c	599 (62%)	80 (61%)	222 (66%)	297 (60%)
Missing	1 (< 1%)	1 (1%)	0 (0%)	0 (0%)
ECOG PS				
0	639 (66%)	61 (46%)	230 (68%)	348 (71%)
> 0	317 (33%)	71 (54%)	107 (32%)	139 (28%)
Missing	6 (1%)	0 (0%)	0 (0%)	6 (1%)
Weight (kg)				
Median (IQR)	78 (66–91)	76 (65–92)	79 (66–89)	78 (67–92)
Missing	7 (1%)	0 (0%)	4 (1%)	3 (1%)

*ECOG PS* Eastern Cooperative Oncology Group performance status, *IQR* interquartile range

Of the pre-treatment characteristics assessed, sex (*P* < 0.001) and body weight (*P* = 0.002) were significantly associated with severe rash (Table 2). Specifically, females (22% risk) were identified as having approximately twice the incidence (OR 2.17; 95% CI 1.52 to 3.09) of severe rash as males (11% risk). Participants with low body weight (< 66 kg, 22% risk) were identified as being at higher risk than the three higher weight groups (9 to 16% risk). In a multivariable analysis including both sex and body weight, only the association between sex and risk of severe rash was statistically significant (*P* = 0.004).

**Table 2:** Univariable association between patient characteristics and risk of severe (grade 3 or 4) rash for patients using vemurafenib alone or in combination with cobimetinib for advanced melanoma

	Events/Patients (%)	OR	95% CI	*P*-value
Sex				< 0.001
Male	64/565 (11%)	1.00		
Female	86/397 (22%)	2.17	1.52 to 3.09	
Age (years)				0.462
< 50	46/345 (13%)	1.00		
50 to 59	42/253 (17%)	1.31	0.83 to 2.07	
60 to 69	39/219 (18%)	1.42	0.89 to 2.27	
≥ 70	23/145 (16%)	1.24	0.72 to 2.15	
ECOG PS				0.597
0	102/639 (16%)	1.00		
1+	47/317 (15%)	0.90	0.61 to 1.32	
Weight (kg)				0.002
< 66	52/233 (22%)	1.00		
66–78	38/242 (16%)	0.64	0.40 to 1.02	
79–90	23/247 (9%)	0.36	0.21 to 0.62	
≥ 91	36/233 (15%)	0.63	0.39 to 1.01	
Body mass index (kg/m^2^)				0.385
18.5–25.0	64/345 (19%)	1.00		
< 18.5	3/21 (14%)	0.74	0.21 to 2.59	
25.1–29.9	47/340 (14%)	0.71	0.47 to 1.08	
≥ 30.0	33/226 (15%)	0.75	0.47 to 1.18	
eGFR (ml/min/1.73 m^2^)				0.228
> 90	71/517 (14%)	1.00		
60–89	67/388 (17%)	1.30	0.91 to 1.88	
45–59	8/41 (20%)	1.46	0.64 to 3.30	
30–44	4/13 (31%)	2.81	0.84 to 9.38	
Bilirubin				0.225
≤ ULN	146/913 (16%)	1.00		
> ULN	3/36 (8%)	0.48	0.14 to 1.58	
AST				0.901
≤ ULN	132/843 (16%)	1.00		
> ULN	16/103 (16%)	0.96	0.55 to 1.70	
ALT				0.108
≤ ULN	138/843 (16%)	1.00		
> ULN	11/106 (10%)	0.58	0.30 to 1.12	
History of atopy				0.279
No	125/767 (16%)	1.00		
Yes	22/176 (13%)	0.77	0.48 to 1.24	

*ALT* alanine aminotransferase, *AST* aspartate aminotransferase, *CI* confidence interval, *ECOG PS* Eastern Cooperative Oncology Group performance status, *eGFR* Estimated glomerular filtration rate, *OR* odds ratio, *ULN* upper limit of normal

**Fig. 1:**
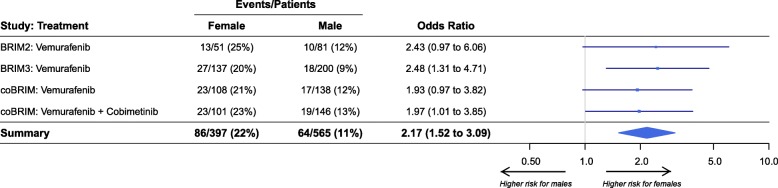
Association between sex and risk of severe (grade 3 or 4) rash stratified by study and treatment

The effect size for the association between sex and risk of severe rash was consistent (Fig. 1) between all studies (BRIM-2, BRIM-3, coBRIM) and treatments (vemurafenib monotherapy, vemurafenib plus cobimetinib). Sex was also significantly associated with the risk of rash classified as a serious adverse event (OR 2.94; 95% CI 1.72 to 7.38; females 3.5% vs males 1.2%).

## Discussion

This pooled analysis of patient-level clinical trial data demonstrates for the first time that patient sex is a significant independent baseline predictor of severe rash occurring with vemurafenib (monotherapy or in combination with cobimetinib) treatment of advanced melanoma. The results of the study indicate that females are twice as likely to develop severe rash with use of vemurafenib therapy.

Cutaneous toxicities are common with use of a BRAF inhibitor or a BRAF-MEK inhibitor combination. Therefore, it is recommended that patients on these treatments undergo monthly to three monthly dermatological reviews to identify and promptly manage dermatological toxicities [14]. Severe rash is one of the most clinically significant treatment-associated cutaneous toxicities, having a negative effect on patients’ quality of life and often requiring vemurafenib dose reduction or temporary/permanent discontinuation [3, 10, 14]. Notably, rash can have a sudden onset and often develops within the first weeks of treatment. The results presented here indicate that it is particularly important for female patients treated with vemurafenib or vemurafenib plus cobimetinib therapy to have comprehensive dermatological education and surveillance to detect and manage rash events, especially in the first several weeks of the treatment. The results presented here relate specifically to treatment involving use of vemurafenib and a future research direction will be to evaluate whether sex is also a predictor of rash adverse events for patients treated with alternative BRAF inhibitors and BRAF-MEK inhibitor combinations.

While our study has highlighted patient sex to be significantly associated with severe rash and its related outcomes, the underlying biological mechanism by which BRAF inhibitors cause rash, and the mechanism by which sex influences the risk of rash are not well understood. It has been hypothesised that BRAF inhibitor induced cutaneous toxicities such as squamous cell carcinoma and keratoacanthoma are caused by keratinocyte proliferation facilitated by the inhibition of wild-type BRAF keratinocytes in the presence of activating RAS mutations, leading to paradoxical activation of MAPK pathway [15–17]. Notably the addition of MEK inhibitor (cobimetinib) therapy to vemurafenib results in marked reduction in risk of squamous cell carcinoma and keratoacanthoma but not rash, which suggests that there are important differences in the mechanisms associated with rash.

The influence of sex on rash may be partly mediated by differences in vemurafenib exposure (plasma concentration) between males and females. It has been reported that following grade ≥ 3 rash resolution, reintroduction of vemurafenib at a lower dose has a low risk of subsequent severe rash [1, 18], and that patients with grade ≥ 2 rash have higher vemurafenib concentration adjacent to the development of rash in comparison to patients without rash [19]. This suggests that higher vemurafenib exposure may be associated with risk of rash. Pharmacokinetic analyses have identified a modest sex based differences in vemurafenib exposure (i.e. AUC and Cmax) with females achieving 14% higher mean steady state vemurafenib exposure than males [20]. This modest increase in vemurafenib exposure may contribute to the increased risk of rash in females. It is possible that there are sex-related differences in the mechanism by which vemurafenib induces rash. However, identifying these contributing factors is limited by the poor current understanding of the mechanism underlying vemurafenib-induced rash.

There has been very limited study to date with respect to baseline predictors of vemurafenib or vemurafenib and cobimetinib associated severe rash. Prior analysis of a small (*n* = 59) cohort of patients treated with vemurafenib monotherapy (predominantly as second or third line therapy) in France reported only an ECOG score of 1 or higher as a predictor of higher risk of grade ≥ 2 rash [19]. Notably, the current study had a much larger sample size (*n* = 962), focused on more severe (grade ≥ 3) rash, evaluated mainly first-line vemurafenib use, included patients from a wider geographical area (primarily North America, Western Europe and Australia/New Zealand) and included patients using vemurafenib in combination with cobimetinib. No association with ECOG performance status was identified in the analysis reported here. However, the data used in this study was limited by the inclusion criteria of the clinical trials which selected only participants with an ECOG performance status of 0 or 1, whereas 15 (25%) individuals in the Kramkimel et al [19] study had an ECOG status of 2 or more. Patients with performance status ≥2 are likely to be more limited in ability to perform daily living activities and are likely to have more progressed disease, factors which may increase the risk of experiencing treatment related toxicities [21]. It is acknowledged that future studies should further investigate variables such as ECOG performance status ≥2, prior antibiotic use, HLA-type, non-White race, and nuances to skin type, as these variables were not available in this unplanned post hoc analysis but may be potential predictors of BRAF inhibitor induced rash. Future studies will also have a role in investigating if the observed increase in the likelihood of severe rash for females with vemurafenib therapy is observed in real-world clinical cohorts. It is of interest to quantify the effect of access to dermatologists, who may have varying opinions on rash severity to oncologists.

## Conclusions

For patients using vemurafenib for the treatment of advanced melanoma, female sex was associated with an approximately two-fold increase in the risk of severe rash and rash classified as a serious adverse event. The association between sex and risk of severe rash was consistent across clinical studies, line of therapy, and the use of vemurafenib therapy alone or in combination with cobimetinib.

## Data Availability

Individual-participant data utilised in this study is available for request to access at clinicalstudydatarequest.com. This study accessed available individual-participant data and clinical study reports from clinical trials NCT00949702 (BRIM-2), NCT01006980 (BRIM-3) and NCT01689519 (coBRIM).

## Abbreviations

ALT: Alanine aminotransferase
AST: Aspartate aminotransferase
BMI: Body mass index
eGFR: Estimated glomerular filtration rate
OR: Odds ratio

## References

[1] Chapman PB, Hauschild A, Robert C, Haanen JB, Ascierto P, Larkin J, Dummer R, Garbe C, Testori A, Maio M (2011). Improved survival with Vemurafenib in melanoma with BRAF V600E mutation. N Engl J Med.

[2] Hauschild A, Grob JJ, Demidov LV, Jouary T, Gutzmer R, Millward M, Rutkowski P, Blank CU, Miller WH, Kaempgen E (2012). Dabrafenib in BRAF-mutated metastatic melanoma: a multicentre, open-label, phase 3 randomised controlled trial. Lancet.

[3] Ascierto PA, McArthur GA, Dréno B, Atkinson V, Liszkay G, Di Giacomo AM, Mandalà M, Demidov L, Stroyakovskiy D, Thomas L (2016). Cobimetinib combined with vemurafenib in advanced BRAFV600-mutant melanoma (coBRIM): updated efficacy results from a randomised, double-blind, phase 3 trial. Lancet Oncol.

[4] Long GV, Stroyakovskiy D, Gogas H, Levchenko E, de Braud F, Larkin J, Garbe C, Jouary T, Hauschild A, Grob JJ (2014). Combined BRAF and MEK inhibition versus BRAF inhibition alone in melanoma. N Engl J Med.

[5] Dummer R, Ascierto PA, Gogas HJ, Arance A, Mandala M, Liszkay G, Garbe C, Schadendorf D, Krajsova I, Gutzmer R (2018). Encorafenib plus binimetinib versus vemurafenib or encorafenib in patients with BRAF -mutant melanoma (COLUMBUS): a multicentre, open-label, randomised phase 3 trial. Lancet Oncol.

[6] Gençler Bilgen, Gönül Müzeyyen (2016). Cutaneous Side Effects of BRAF Inhibitors in Advanced Melanoma: Review of the Literature. Dermatology Research and Practice.

[7] Rinderknecht JD, Goldinger SM, Rozati S, Kamarashev J, Kerl K, French LE, Dummer R, Belloni B (2013). RASopathic skin eruptions during Vemurafenib therapy. PLoS One.

[8] Mandalà M, Massi D, De Giorgi V (2013). Cutaneous toxicities of BRAF inhibitors: clinical and pathological challenges and call to action. Crit Rev Oncol Hematol.

[9] McArthur GA, Chapman PB, Robert C, Larkin J, Haanen JB, Dummer R, Ribas A, Hogg D, Hamid O, Ascierto PA (2014). Safety and efficacy of vemurafenib in BRAF(V600E) and BRAF(V600K) mutation-positive melanoma (BRIM-3): extended follow-up of a phase 3, randomised, open-label study. Lancet Oncol.

[10] Dréno B, Ribas A, Larkin J, Ascierto PA, Hauschild A, Thomas L, Grob JJ, Koralek DO, Rooney I, Hsu JJ (2017). Incidence, course, and management of toxicities associated with cobimetinib in combination with vemurafenib in the coBRIM study. Ann Oncol.

[11] Sosman JA, Kim KB, Schuchter L, Gonzalez R, Pavlick AC, Weber JS, McArthur GA, Hutson TE, Moschos SJ, Flaherty KT (2012). Survival in BRAF V600–mutant advanced melanoma treated with Vemurafenib. N Engl J Med.

[12] https://clinicalstudydatarequest.com/Study-Sponsors/Study-Sponsors-Roche.aspx. Accessed 19 July 2019.

[13] https://www.fda.gov/safety/reporting-serious-problems-fda/what-serious-adverse-event. Accessed 19 June 2019.

[14] Lacouture ME, Duvic M, Hauschild A, Prieto VG, Robert C, Schadendorf D, Kim CC, McCormack CJ, Myskowski PL, Spleiss O (2013). Analysis of dermatologic events in vemurafenib-treated patients with melanoma. Oncologist.

[15] Hatzivassiliou G, Song K, Yen I, Brandhuber BJ, Anderson DJ, Alvarado R, Ludlam MJC, Stokoe D, Gloor SL, Vigers G (2010). RAF inhibitors prime wild-type RAF to activate the MAPK pathway and enhance growth. Nature.

[16] Anforth RM, Blumetti TCMP, Kefford RF, Sharma R, Scolyer RA, Kossard S, Long GV, Fernandez-Peñas P (2012). Cutaneous manifestations of dabrafenib (GSK2118436): a selective inhibitor of mutant BRAF in patients with metastatic melanoma. Br J Dermatol.

[17] Su F, Viros A, Milagre C, Trunzer K, Bollag G, Spleiss O, Reis-Filho JS, Kong X, Koya RC, Flaherty KT (2012). RAS mutations in cutaneous squamous-cell carcinomas in patients treated with BRAF inhibitors. N Engl J Med.

[18] Sinha R, Edmonds K, Newton-Bishop JA, Gore ME, Larkin J, Fearfield L (2012). Cutaneous adverse events associated with vemurafenib in patients with metastatic melanoma: practical advice on diagnosis, prevention and management of the main treatment-related skin toxicities. Br J Dermatol.

[19] Kramkimel N, Thomas-Schoemann A, Sakji L, Golmard J, Noe G, Regnier-Rosencher E, Chapuis N, Maubec E, Vidal M, Avril M (2016). Vemurafenib pharmacokinetics and its correlation with efficacy and safety in outpatients with advanced BRAF-mutated melanoma. Target Oncol.

[20] Zhang W, Heinzmann D, Grippo JF (2017). Clinical pharmacokinetics of Vemurafenib. Clin Pharmacokinet.

[21] Oken MM, Creech RH, Tormey DC, Horton J, Davis TE, McFadden ET, Carbone PP (1982). Toxicity and response criteria of the eastern cooperative oncology group. Am J Clin Oncol.

